# Public Perception Analysis of Tweets During the 2015 Measles Outbreak: Comparative Study Using Convolutional Neural Network Models

**DOI:** 10.2196/jmir.9413

**Published:** 2018-07-09

**Authors:** Jingcheng Du, Lu Tang, Yang Xiang, Degui Zhi, Jun Xu, Hsing-Yi Song, Cui Tao

**Affiliations:** ^1^ School of Biomedical Informatics The University of Texas Health Science Center at Houston Houston, TX United States; ^2^ Department of Communication College of Liberal Arts Texas A&M University College Station, TX United States

**Keywords:** convolutional neural networks, social media, measles, public perception

## Abstract

**Background:**

Timely understanding of public perceptions allows public health agencies to provide up-to-date responses to health crises such as infectious diseases outbreaks. Social media such as Twitter provide an unprecedented way for the prompt assessment of the large-scale public response.

**Objective:**

The aims of this study were to develop a scheme for a comprehensive public perception analysis of a measles outbreak based on Twitter data and demonstrate the superiority of the convolutional neural network (CNN) models (compared with conventional machine learning methods) on measles outbreak-related tweets classification tasks with a relatively small and highly unbalanced gold standard training set.

**Methods:**

We first designed a comprehensive scheme for the analysis of public perception of measles based on tweets, including 3 dimensions: discussion themes, emotions expressed, and attitude toward vaccination. All 1,154,156 tweets containing the word “measles” posted between December 1, 2014, and April 30, 2015, were purchased and downloaded from DiscoverText.com. Two expert annotators curated a gold standard of 1151 tweets (approximately 0.1% of all tweets) based on the 3-dimensional scheme. Next, a tweet classification system based on the CNN framework was developed. We compared the performance of the CNN models to those of 4 conventional machine learning models and another neural network model. We also compared the impact of different word embeddings configurations for the CNN models: (1) Stanford GloVe embedding trained on billions of tweets in the general domain, (2) measles-specific embedding trained on our 1 million measles related tweets, and (3) a combination of the 2 embeddings.

**Results:**

Cohen kappa intercoder reliability values for the annotation were: 0.78, 0.72, and 0.80 on the 3 dimensions, respectively. Class distributions within the gold standard were highly unbalanced for all dimensions. The CNN models performed better on all classification tasks than k-nearest neighbors, naïve Bayes, support vector machines, or random forest. Detailed comparison between support vector machines and the CNN models showed that the major contributor to the overall superiority of the CNN models is the improvement on recall, especially for classes with low occurrence. The CNN model with the 2 embedding combination led to better performance on discussion themes and emotions expressed (microaveraging F1 scores of 0.7811 and 0.8592, respectively), while the CNN model with Stanford embedding achieved best performance on attitude toward vaccination (microaveraging F1 score of 0.8642).

**Conclusions:**

The proposed scheme can successfully classify the public’s opinions and emotions in multiple dimensions, which would facilitate the timely understanding of public perceptions during the outbreak of an infectious disease. Compared with conventional machine learning methods, our CNN models showed superiority on measles-related tweet classification tasks with a relatively small and highly unbalanced gold standard. With the success of these tasks, our proposed scheme and CNN-based tweets classification system is expected to be useful for the analysis of tweets about other infectious diseases such as influenza and Ebola.

## Introduction

Nearly 40 million cases of measles, caused by a highly contagious virus, lead to over 300,000 deaths worldwide every year [1]. In the United States, measles was officially declared to be eliminated in 2000 thanks to the successful nationwide administration of a 2-dose vaccination program [2]. However, recent years have seen the reemergence of measles outbreaks in the United States. The most recent large-scale measles outbreak occurred in early 2015 with a high concentration of cases in California [3]. Researchers believe that increasing rates of vaccination refusal and undervaccination have made the public more vulnerable to this potentially deadly disease [4].

During an outbreak of an infectious disease such as measles, responsible public health agencies need to send out timely messages to the public during different stages of the crisis [5]. For instance, the Centers for Disease Control and Prevention (CDC) has adopted a 5-stage model of crisis and emergency risk communication, including precrisis, initial event, maintenance, resolution, and evaluation [5]. Prompt understanding of the public’s perceptions will allow public health agencies to respond to people’s attitudes, emotions, and needs in real time instead of relying on a predetermined timeline based on stages. Using traditional methods such as surveys to study public perceptions during an infectious disease outbreak is both costly and time-consuming [4,6].

Social media have been increasingly used by the general public, patients, and health professionals to communicate about health-related issues [7]. Researchers have studied social media content for drug adverse events detection [8,9], assessment of public opinion about health-related issues such as vaccination [10-13], and infectious disease outbreak surveillance [6,14,15]. Twitter, one of the largest public social media in the world, provides unique insights into how the public responds to an infectious disease outbreak as users, in real time, share information about the outbreak, talk about their personal experiences, argue over the necessity and safety of vaccination, and express a wide range of emotions. Examining Twitter content can provide an immediate assessment of the public’s response and will allow public health professionals to adapt their messages to communicate with the public more effectively.

Many studies have used Twitter to assess various public health topics. However, most of the studies thus far have focused on analyzing the frequency of postings rather than on understanding post contents [16]. There is an increasing need to develop automatic and scalable approaches for the accurate understanding of the high volume of Twitter posts. Recent advances in machine learning and natural language processing (NLP) technologies allow for the stringent analysis of large amounts of Twitter posts. However, compared to texts in other domains, Twitter text has very distinctive characteristics such as very short text, unique Twitter language and structures, etc. For some health-related topics, there also exists the unbalanced class distribution issue (certain classes are much more frequent than other classes), which can further erode the performance of NLP models [10,13]. To improve performance on health-related Twitter datasets, substantial time and effort on feature engineering [10,17,18] is needed for conventional machine-learning algorithms, including support vector machines (SVMs), k-nearest neighbors (KNNs), etc.

Compared to conventional machine learning algorithms, neural network models are advantageous because they have saved significant time on task-specific features engineering, achieved higher performance, and are scalable to large applications [19]. Some recent works applied neural network models to social media to understand public perceptions and behaviors. For instance, Lima et al [20] investigated the use of a multilayer perceptron neural network to classify personality from Twitter. Huynh et al [21] and Coco et al [22] proposed a deep neural network model to identify adverse drug reactions from Twitter data. Kendra [23] used a 5-layer neural network to characterize the discussion about antibiotics on Twitter. Bian et al [24] applied a convolutional neural network model to perform sentiment analysis on layperson’s tweets. Zhao et al [25] proposed a semisupervised deep learning for influenza epidemic simulation. However, to our best knowledge, little work has been done to study public perceptions of infectious diseases and vaccinations on Twitter using neural network models.

## Methods

### Data Collection

**Figure 1:**
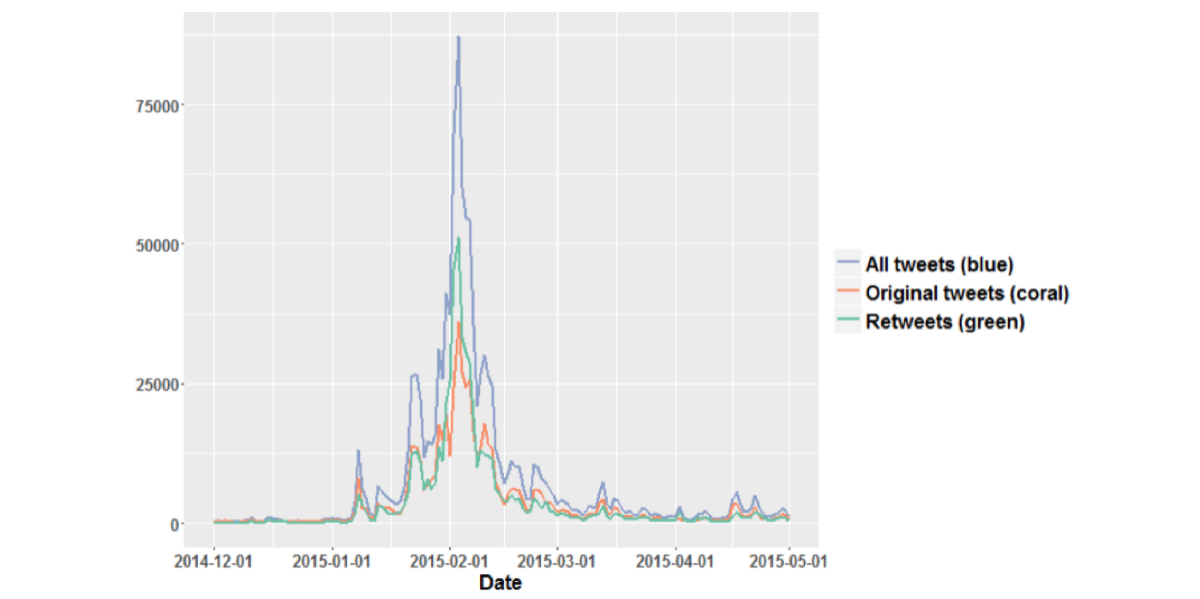
Frequency of measles-related tweets by date and type.

All tweets including the word “measles” posted between December 1, 2014, and April 30, 2015, were purchased and downloaded from DiscoverText.com. This time frame was chosen because the unidentified Patient Zero of this outbreak visited the Disneyland theme park in California in December 2014. The first few suspected cases of measles were reported on January 5, 2015, and the last case was reported on March 2, 2015. CDC officially declared the outbreak to be over on April 17, 2015 [26]. A total of 1,154,156 tweets were collected. The number of tweets collected during the time frame can be seen in Figure 1.

### Gold Standard Annotation

**Figure 2:**
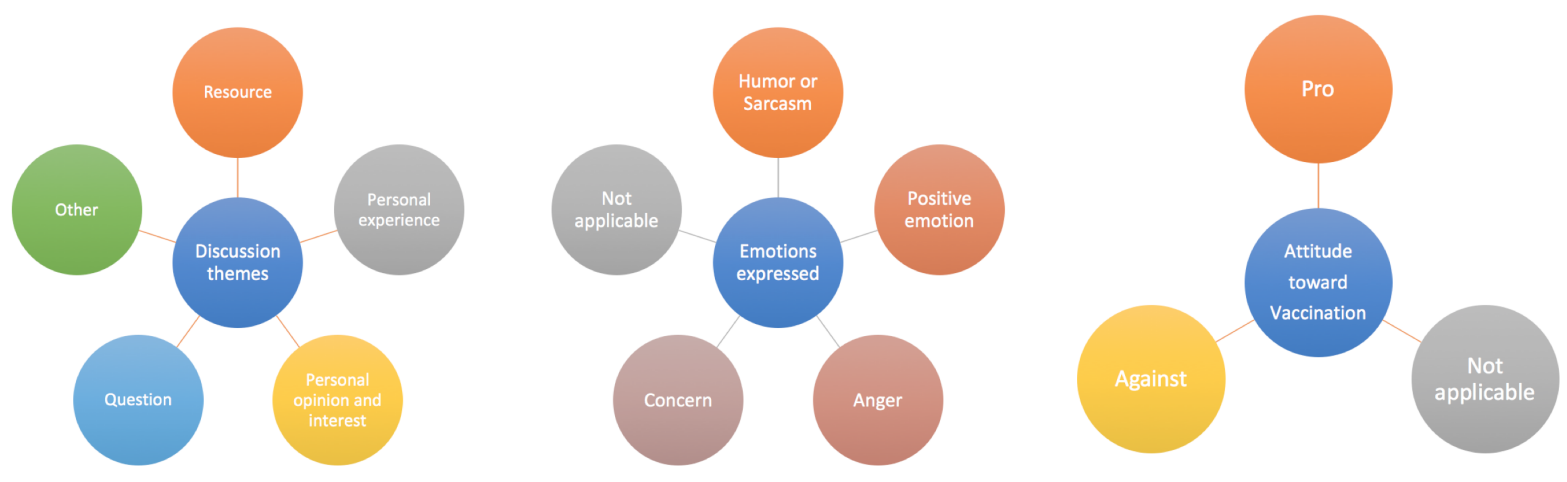
Measles tweets annotation scheme for different dimensions.

In order to understand measles-related contents on Twitter comprehensively, we created an annotation scheme containing 3 dimensions: *discussion themes*, *emotions expressed*, and *attitude toward vaccination*. The coding schemes *discussion themes* and *emotions expressed* were adapted based on Chew and Eysenbach [6], while the coding scheme *attitude toward vaccination* was created by the authors inductively. For *discussion themes*, 5 themes were identified: resources (news update about the outbreak, medical information about prevention, treatment, symptoms of measles), personal experience (direct or indirect experiences about measles), personal opinions and interests, questions, and other (unrelated to measles). *Emotions expressed* was categorized into 5 types: humor or sarcasm, positive emotion (relief and downplayed risk), anger, concern, and not applicable. The data collection was based on the keyword measles; however, debate about vaccines emerged in a large percentage of tweets collected. Hence, we took this opportunity to measure how public opinion changed over time during a measles outbreak. *Attitude toward vaccination* was categorized into 3 groups: pro (provaccination), against (antivaccination), and not applicable (no attitude). See Figure 2 for a visual representation of the 3 dimensions and categories within each dimension.

Two coders manually coded 0.1% of all tweets selected through systematic sampling. The first tweet was identified using a random number generator. After this, every 1000th tweet was selected in the sample. The Cohen kappa intercoder reliability values for the 3 dimensions were 0.78, 0.72, 0.80, respectively. Afterward, the 2 coders discussed their results to resolve discrepancies.

### Neural Network Classification System

#### Data Cleaning

The vocabulary used on Twitter is very different from the general English vocabulary. User names, URLs, and hashtags need to be normalized. We first replaced tokens containing all capital letters with the lowercase of the token with string “<ALLCAPS>”. Then all URLs were replaced with string “<URL>”. Twitter user names (eg, @twitter) were then replaced with string “<USER>”. All numbers were replaced with string “<NUMBER>”. All hashtags were separated into tokens by uppercase letters (eg, we replace “#VaccineWork” with “<HASHTAG> Vaccine Work”). Afterwards, all tweets were converted to lowercase. Our tweets preprocessing process was based on the Stanford GloVe tweets preprocessing script [27]. An example illustrating the tweet preprocessing step is shown below:

Raw tweet text: “RT @KTLA: #BREAKING: At least 9 measles cases linked to visits to @Disneyland from Dec. 15-20 http://t.co/1GRlwFhPgv http://t.co/3Nl15jmqAE”

Cleaned tweet text: “rt <allcaps> <user>: breaking: at least <number> measles cases linked to visits to <user> from dec. <number> <number> <url> <url>”

#### Convolutional Neural Networks

**Figure 3:**
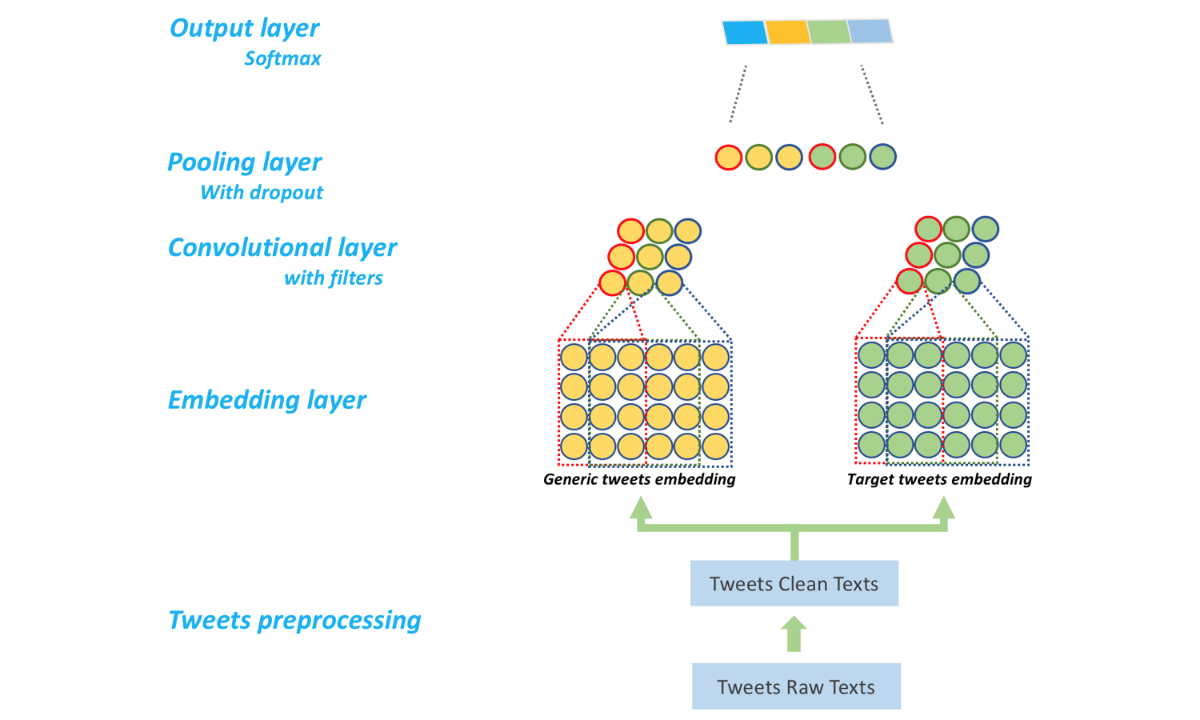
System architecture for measles-related tweets classification using convolutional neural networks.

Commonly used in various computer vision tasks [28], convolutional neural networks (CNNs) have demonstrated excellent performance in the NLP field, including different text classification tasks [29-32]. We extended the classic CNN framework for sentence classification proposed by Kim [29] by using combination generic Twitter embedding and target domain Twitter embedding [33]. Details of our CNN system architecture can be seen in Figure 3. We cleaned the tweets following the data cleaning step. Then each token of the tweets was mapped to 2 high-dimension representations through 2 word embeddings: generic tweets embedding and target domain tweets embedding. Both embeddings were fine-tuned during the training process.

We used 3 filters of size 3, 4, and 5 to generate the convolutional layer on each embedding. The feature maps generated by filters from each embedding were concatenated and fed to the pooling layer. We adopted max-pooling strategy with a dropout rate at 0.5 on the pooling layer. The output layer consisted of different classes for each dimension. This CNN system was built based on the Python and Tensorflow libraries [34].

#### Tweets Word Vector Embedding

For generic tweets embedding, we used pretrained GloVe tweets embedding from Stanford. GloVe is an unsupervised learning algorithm developed by Pennington et al [35] to obtain vector representations for words. GloVe tweets word vectors were trained on 2 billion tweets and 27 billion tokens [35] and have been widely used in different Twitter-related NLP tasks [31,36,37]. For target domain embedding, we trained a tweets embedding from our own measles-related tweets corpus (1,154,156 tweets) using the same GloVe algorithm. We tested different numbers of embedding dimensions in our preexperiments. The tweets word embedding in dimension 200 achieved the best performance for our tasks.

### Experiments

For the CNN-based framework, we performed the following experiments: (1) use of pretrained GloVe tweets embedding only, (2) use of tweets measles embedding only, and (3) use of a combination of the pretrained GloVe tweets embedding and measles tweets embedding. For the use of 1 embedding only, we just used 1 channel of the proposed framework. We chose 4 popular machine learning models for comparison as our baselines: KNN [38], naïve Bayes [39], SVM [40], and random forest [41]. For SVM, a radial basis function kernel was used. We followed the same tweet cleaning steps and extracted n-grams as the feature for these traditional machine learning models. The Waikato Environment for Knowledge Analysis library was used to train and test these models [42]. We also evaluated the bidirectional long short-term memory (Bi-LSTM), which has achieved state-of-the-art performance in many classification and sequence labeling tasks [43,44], for tweets classifications. The input of the Bi-LSTM is the pretrained GloVe tweets embedding (dimension: 200). We conducted these experiments on all 3 dimensions for public perceptions on measles.

### System Evaluation

We leveraged a 10-fold cross-validation to evaluate the performances of these models for each classification task. Standard metrics including precision, recall, and F1 score were calculated for each class. We also calculated the microaveraging F score and macroaveraging F score to evaluate their performance on each classification task. For microaveraged score, we summed up all the individual true positives, false positives, and false negatives. For macroaveraged score, we took the average of the F1 score of different categories.

### Ethical Approval

This study received institutional review board approval from the Committee for the Protection of Human Subjects at the University of Texas Health Science Center at Houston. The reference number is HSC-SBMI-16-0291.

## Results

### Gold Standard Description

In total, 1151 tweets were annotated. Class distributions were highly unbalanced for all 3 tasks (Table 1). In terms of *discussion themes*, nearly two-thirds (718/1151, 62.38%) of tweets were categorized as resources (ie, outbreak update or medical information about measles). Less than one-third (344/1151, 29.89%) of the tweets were about users’ personal opinions and interests. Only 1.82% (21/1151) of the tweets discussed personal experience with measles, and 1.73% (20/1151) asked questions. For *emotions expressed*, 79.84% (919/1151) of tweets were categorized as expressing concern. Humor or sarcasm was found in 9.47% (109/1151) of the tweets. Positive emotion and anger were found in 3.38% (39/1151) and 3.04% (35/1151) of the tweets, respectively. Finally, in terms of *attitude toward vaccination*, the majority of the tweets (913/1151, 79.32%) did not express any opinion about vaccination, 17.55% (202/1151) of tweets were provaccination and 3.13% (36/1151) were antivaccination.

### Overall Comparison of Convolutional Neural Network Models With Conventional Models

Comparison of the performances of CNN models and 4 machine learning models on the 3 dimensions can be seen in Table 2. As shown, CNN-based models have better performance than other conventional machine learning models or the Bi-LSTM model. The CNN model with the combination of 2 embeddings achieved the best performance on *emotions expressed* and the highest macroaveraging F score on *discussion themes*. The CNN model with Stanford embedding had the highest microaveraging F score on *discussion themes* and achieved the best performance on *attitude toward vaccination*. The CNN with measles embedding achieved relatively high microaveraging F score on *emotions expressed* and *attitude toward vaccination*. The Bi-LSTM model had the worst performance among neural network models, probably due to the limited size of training data.

**Table 1:** Class distribution in the gold standard for 3 dimensions.

Dimension and class			Tweets, n (%)
**Discussion themes**			
	Resource	718 (62.4)	
	Personal experience	21 (1.8)	
	Personal opinions and interest	344 (29.9)	
	Question	20 (1.7)	
	Other	48 (4.2)	
**Emotions expressed**			
	Humor or sarcasm	109 (9.5)	
	Positive emotion	39 (3.4)	
	Anger	35 (3.0)	
	Concern	919 (79.8)	
	Not applicable	49 (4.3)	
**Attitude toward vaccination**			
	Pro	202 (17.6)	
	Against	36 (3.1)	
	Not applicable	913 (79.3)	

**Table 2:** Ten-fold cross-validation results of neural network models and 4 conventional machine learning models on 3 dimensions. Italics indicate best performance in that class.

Model	Microaveraging F score			Macroaveraging F score		
	Discussion themes	Emotions expressed	Attitude toward vaccination	Discussion themes	Emotions expressed	Attitude toward vaccination
KNN^a^	0.5143	0.6977	0.8129	0.3223	0.4074	0.5114
Naïve Bayes	0.6811	0.7767	0.7171	0.4101	0.4814	0.5343
Random forest	0.7350	0.8393	0.8085	0.4243	0.4393	0.5356
SVM^b^	0.7696	0.8365	0.8211	0.3917	0.4269	0.5345
Bi-LSTM^c^	0.7315	0.8271	0.7958	0.2899	0.3730	0.4358
CNN_M^d^	0.7533	0.8480	0.8355	0.4282	0.4849	0.5871
CNN_S^e^	*0.7897*	0.8575	*0.8642*	0.4158	0.5419	*0.6629*
CNN_M+S^f^	0.7811	*0.8592*	0.8254	*0.4611*	*0.5591*	0.6078

^a^KNN: k-nearest neighbor.

^b^SVM: support vector machines.

^c^Bi-LSTM: bidirectional long short-term memory.

^d^CNN_M: convolutional neural network using the measles tweets embedding.

^e^CNN_S: convolutional neural network using the pretrained GloVe tweets embedding from Stanford.

^f^CNN_M+S: convolutional neural network using the combination of pretrained GloVe tweets embedding and measles tweets embedding.

As shown in Table 2, among the conventional machine learning models, SVM generally performed the best on all 3 dimensions. In order to further compare the performances of CNN models on each class and try to improve the overall performance, we then calculated and compared the precision, recall, and F score of SVM, the CNN model with Stanford GloVe tweets embedding only, and the CNN model with the combination of generic and target domain embedding.

### Detailed Comparison of Convolutional Neural Network Models With Support Vector Machines on 3 Dimensions

Table 3 shows the comparison of SVM and CNN models on *discussion themes*. For precision score, the CNN with GloVe tweets embedding achieved better performance on classes with larger numbers of tweets (resources and personal opinions and interest). The CNN with the combination of 2 embeddings achieved better performance on classes with very limited numbers of tweets (ie, questions). For recall score, the CNN model with either Stanford embedding or the combination of 2 embeddings greatly improved the recall of the classes with relatively fewer tweets such as personal opinions and interests and questions, while SVM had slightly better performance on resources. The improvement of recall score greatly contributed to the improvement on the F score. Unfortunately, for the class personal experience, none of the models could identify any tweets correctly.

The comparison of SVM and the CNN models on *emotions expressed* can be seen in Table 4. CNN models achieved higher precision scores on classes with fewer cases, including anger and not applicable, while SVM performed better on humor or sarcasm. For recall and F1 score, CNN models with either Stanford embedding or the combination of 2 embeddings performed well on all classes. In general, the CNN with the combination of 2 embeddings had better performance for more categories than the CNN with Stanford embedding only.

For dimension 3, *attitude toward vaccination*, the overall comparison between the CNN models and SVM can be seen in Table 5. Both CNN models outperformed SVM in most of the categories, and the CNN model with Stanford embedding achieved better performance in most of the categories. Specifically, for precision score, SVM performed better on class pro, while the CNN models did better on class against and not applicable. The CNN with the combination of 2 embeddings achieved the highest precision score on against. In terms of recall, the CNN models performed much better on the classes with very small numbers of tweets (ie, pro and against), while SVM did better on the class not applicable. As for F1 score, the CNN with Stanford embedding performed the best, and SVM performed the worst on all 3 classes.

**Table 3:** Detailed precision, recall, and F score of each class for *discussion themes*. Italics indicate best performance in that class.

Class	Precision			Recall			F1 score			
	SVM^a^	CNN_M+S^b^	CNN_S^c^	SVM	CNN_M+S	CNN_S		SVM	CNN_M+S	CNN_S
Resource (n=718)	0.7907	0.8119	*0.8172*	*0.9471*	0.9318	0.9401		0.8619	0.8677	*0.8744*
Personal experience (n=21)	0	0	0	0	0	0		0	0	0
Personal opinions and interest (n=344)	0.7021	0.6984	*0.7231*	0.5773	0.6192	*0.6453*		0.6336	0.6564	*0.6820*
Question (n=20)	0	0.5	0	0	0.0500	0		0	*0.0909*	0
Other (n=48)	0.8750	0.8421	*0.8571*	0.1458	*0.3333*	0.2500		0.2500	*0.4776*	0.3871

^a^SVM: support vector machines.

^b^CNN_M+S: convolutional neural network using the combination of pretrained GloVe tweets embedding and measles tweets embedding.

^c^CNN_S: convolutional neural network using the pretrained GloVe tweets embedding from Stanford.

**Table 4:** Detailed precision, recall and F scores of each class for *emotions expressed*. Italics indicate best performance in that class.

Class	Precision			Recall					F1 score			
	SVM^a^	CNN_M+S^b^	CNN_S^c^		SVM		CNN_M+S	CNN_S		SVM	CNN_ M+S	CNN_S
Humor or sarcasm (n=109)	*1*	0.9388	0.8909		0.3486	0.4220		*0.4495*		0.5170	0.5823	*0.5976*
Positive emotion (n=39)	*1*	*1*	*1*		0.0513	*0.1538*		0.1282		0.0967	*0.2667*	0.2273
Anger (n=35)	0	*1*	0.6667		0	0.0286		*0.0571*		0	0.0556	*0.1053*
Concern (n=919)	0.8312	0.8538	*0.8550*		0.9069	*0.9978*		0.9946		0.9069	*0.9202*	0.9195
Not applicable (n=49)	0.7500	*0.9048*	0.8947		0.2105	*0.3878*		0.3469		0.2105	*0.5429*	0.5000

^a^SVM: support vector machines.

^b^CNN_M+S: convolutional neural network using the combination of pretrained GloVe tweets embedding and measles tweets embedding.

^c^CNN_S: convolutional neural network using the pretrained GloVe tweets embedding from Stanford.

**Table 5:** Detailed precision, recall, and F score of each class for *attitude toward vaccination*. Italics indicate best performance in that class.

Class	Precision			Recall				F1 score			
	SVM^a^	CNN_M+S^b^	CNN_S^c^		SVM	CNN_M+S	CNN_S		SVM	CNN_M+S	CNN_S
Pro (n=202)	*0.7917*	0.6458	0.7554		0.1919	0.3069	*0.5198*		0.3089	0.4161	*0.6158*
Against (n=36)	0.6667	*1*	0.8571		0.0556	*0.1667*	*0.1667*		0.1026	*0.2857*	0.2791
Not applicable (n=913)	0.8228	0.8408	*0.8794*		*0.9890*	0.9660	0.9682		0.8982	0.8991	*0.9216*

^a^SVM: support vector machines.

^b^CNN_M+S: convolutional neural network using the combination of pretrained GloVe tweets embedding and measles tweets embedding.

^c^CNN_S: convolutional neural network using the pretrained GloVe tweets embedding from Stanford.

## Discussion

### Principal Contributions

This study makes 2 primary contributions. First, we designed and implemented a comprehensive scheme for the public perception analysis of measles-related tweets, including *discussion themes*, *emotions expressed*, and *attitude toward vaccination*. We manually curated a gold standard set that contains 1151 tweets annotated according the scheme. The tweets were sampled from all measles-related tweets during the most recent measles outbreak in the United States in 2015. Based on the annotation results, we believe the scheme can successfully classify the public’s opinions and emotions. Second, we designed and implemented CNN models on the classification tasks of measles-related tweets and investigated their performance compared to traditional machine learning models through a comprehensive comparison on the small-scale tweets corpus with highly unbalanced class distribution.

### Principal Findings

In classifying measles-related tweets in terms of *discussion themes*, *emotions expressed*, and *attitude toward vaccination*, different classifiers were better suited for different tasks. However, the CNN models achieved better overall performance on all 3 tasks compared to conventional machine learning algorithms. A detailed comparison of the CNN models and SVM showed that the CNN models were able to improve performance on nearly all classes for all 3 dimensions. The major contributor to the overall performance boost is the improvement on recall, especially for the classes with fewer cases than average. The CNN model with the combinations of 2 embeddings led to better performance on *discussion themes* and *emotions expressed*, while the CNN model with Stanford embedding achieved best performance on *attitude toward vaccination*. A common obstacle of deep neural network-based models is the need for a large training dataset. However, for a disease-related tweets classification task like ours, the results show that CNN models can perform better than conventional machine learning models even on a training dataset with only 1151 labeled tweets.

### Limitations and Future Directions

Although the CNN models can greatly increase the performance for most of the classes with few cases, for some minor classes with extremely low numbers of cases such as personal experience in *discussion themes*, the CNN models are just as powerless as conventional models. Further examination of the prediction results shows that many tweets in the minor classes were incorrectly classified into major classes. For example, the tweets in personal experience were either classified as resources or personal opinions and interest. For against in *attitude toward vaccination*, the majority of the tweets were classified as not applicable, which takes up to 79% of the labeled data. The highly unbalanced class distribution is a major challenge for both conventional machine learning methods and neural network methods. Since the current gold standard training set is relatively small, we plan to collect and annotate more related tweets (especially the tweets belonging to smaller classes) to build a larger labeled dataset. We believe performance could be improved by using a larger labeled training dataset.

Future research could take a few directions. Additional hyperparameter tuning (ie, activation functions selection, pooling strategies) can also improve the performance on the disease-related tweets classification tasks. In addition, although the Bi-LSTM model doesn’t work well on our tasks (probably due to the limited training data size), other recurrent neural network-based frameworks such as attentive Bi-LSTM [45] may lead to better performance, especially as the size of the training data increases. The improved models can be used to automatically predict the labels of the measles tweets, which will facilitate the analysis of large scale public perceptions about measles as well as other infectious diseases. Some unsupervised machine learning methods can also be used to explore the major discussion topics from the measles-related tweets dataset, such as topic modeling methods [46,47], as it can save the effort of annotation.

### Conclusion

Timely understanding of public perceptions during the outbreak of an infectious disease such as measles will allow public health agencies to adapt their messages to address the needs, concerns, and emotions of the public. In order to understand the contents of Twitter text regarding measles and vaccination, we designed a classification scheme that contains *discussion themes*, *emotions expressed*, and *attitude toward vaccination* for measles-related tweets. A gold standard containing 1151 tweets was collected and manually annotated according to the classification scheme. CNN models have been evaluated to classify tweets into different classes for different tasks. A comparative study was done to evaluate the performance of CNN models in comparison to 4 conventional machine learning models as well as a Bi-LSTM model. The CNN models had improved performance on classification of themes, emotions, and attitude from the highly unbalanced measles-related tweets dataset. The CNN models presented in the paper can be applied on large-scale tweets datasets. Our proposed scheme and CNN-based tweets classification system for the public perception analysis on Twitter toward measles disease can be used for other infectious diseases such as influenza and Ebola.

## Abbreviations

Bi-LSTM: bidirectional long short-term memory
CNN: convolutional neural networks
KNN: k-nearest neighbors
SVM: support vector machines
CDC: Centers for Disease Control and Prevention
NLP: natural language processing
